# Kin or Research Material? Exploring IVF Couples’ Perceptions about the Human Embryo and Implications for Disposition Decisions in Norway

**DOI:** 10.1007/s11673-022-10214-7

**Published:** 2022-11-04

**Authors:** B. Kvernflaten, P. Fedorcsák, K. N. Solbrække

**Affiliations:** 1 Department for Interdisciplinary Health Sciences, Institute of Health and Society, University of Oslo, Forskningsveien 3a, 0373 Oslo, Norway; 2 Department of Reproductive Medicine, Oslo University Hospital, Sognsvannsveien 20, Oslo, 0372 Norway; 3 Faculty of Medicine, University of Oslo, Oslo, Norway

**Keywords:** IVF, Reproductive technologies, Embryo research, Embryo donation, Liminality, Kinship

## Abstract

In vitro fertilization (IVF) involves making embryos outside of the human body, which has spurred debate about the status of the embryo, embryo research and donation. We explore couples’ perceptions about embryos and their thoughts and acceptability about various disposition decisions in Norway. Based on an ethnographic study including interviews and observations in an IVF clinic, we show that couples do not perceive their pre-implantation IVF embryos to be human lives; rather, they consider successful implantation the start of life. We suggest that this response indicates a change in the perception of the human embryo or the fertilised egg from *incipient life*—a viewpoint that was dominant in the discussions of embryo research in the 1980s and 1990s. We also show how this view of the pre-implantation embryo elucidates why donating embryos to research appears acceptable but donating to other infertile couples seems rather difficult. Before transfer to a woman’s uterus, the embryo exists in a liminality; it is not yet human life but a living cell with potential for both research *and* pregnancy. When an embryo is implanted and pregnancy is confirmed, human life activates; the embryo becomes potential kin, influencing couples’ struggles with donating embryos to other couples.

## Introduction

During the process of in vitro fertilization (IVF), eggs are retrieved from a woman’s body and fertilised in a petri dish in the lab outside of her body, only to be transferred back for pregnancy. Although these technological advances have allowed millions of babies to be born since 1978, they have also raised debates about the moral status of the human embryo, particularly for embryo research and donation. At the heart of the debate have been the controversies and tensions between societal interests in medical advances and worries regarding treating human embryos with dignity (McMahon et al. 2003). The embryo in such a context has been subjected to philosophical, theological, ethical and political debate and scrutiny (Melhuus 2015). The ways in which patients or couples undergoing IVF, who are the ones going through treatment and potentially donating their embryos, relate to their surplus embryos have received increased attention in the global literature. Surplus refers to either fresh embryos sorted by the embryologist in the clinic as unsuitable for implantation into a woman’s’ womb because of poor quality or to cryopreserved (frozen) spare embryos that remain after the couple ends fertility treatment (Parry 2006). Disposal options for cryopreserved embryos involve discarding them, continuing to freeze them, or donating them to research or to another infertile couple. However, the legal frameworks for storage limits of cryopreserved embryos and donation options vary in different countries worldwide.

Millbank et al. (2016) show that it is exactly in such various local contexts and differences that our understanding of patient views and experiences about donation must be situated. Such views, experiences and disposition decisions, they argue, do not occur in a social or legal vacuum but are influenced by a number of external factors (Millbank, Stuhmcke, and Karpin 2016). Goedeke et al. (2017) highlight how choices and actions in relation to embryos are complex; they point to several studies (for instance Nachtigall et al. 2005; de Lacey 2007; Lyerly et al. 2010) showing that decisions—which take place in the context of cultural factors and changing public policy—are emotionally challenging, demand extensive moral reasoning, and are often delayed. Research has also shown how choices made about embryo donation and their future are shaped by notions of kinship, family and relations (Roberts 2011). Kato and Sleeboom-Faulkner (2011) show how cultural notions of motherhood (i.e., the mother’s view about her responsibility towards her children) play a crucial role in the disposal and ontological meaning of embryos. For some, understanding how patients conceptualise the human embryo—its moral status—is central in influencing their disposition choices (see Goedeke et al. 2017). Although the embryo is a global entity in terms of being of the same material across the world, its particular meaning is created in local context (Melhuus 2015). Research conducted on the topic in various countries shows the different meanings attached to embryos in a variety of contexts (Parry 2006).

Franklin (1995) has pointed to how the human embryo made outside of the human body exists in a liminality, a contested location between science and nature. According to Western kinship definitions, the embryo satisfy the criteria of kin relatedness in that it has a genetic link to its parents; however, it is also a microscopic entity made in a lab. It is fully human, but it is also potential research material (Franklin 1995). Building on Franklin’s discussions of liminality, Millbank (2017) explores how women expressed such a contested location as something of-the-body but not within the body, neither self nor other, person nor thing. Ellison and Karpin (2011) discuss an embryo’s liminality as something between life and death through a discussion of “compassionate transfer”, which involves thawing the cryopreserved embryos, placing it in the woman’s vagina or cervix where it cannot develop, or uterus at a time when implantation is unlikely. This ritual emerges to manage the dynamic categories of life and death (Ellison and Karpin 2011). It is such liminality of the embryo that makes embryo research a field of contestations, providing “a template of cultural definitions of what it is to be a person and what makes a kinship tie” (Franklin 1995, 337). Such disputes have manifested in discussions about the embryo as *not life* (in favour of embryo research) and the embryo as *human life* (hence considering embryo research as problematic) (see McMahon et al. 2003; Melhuus 2015), showing the negotiations of the life status of liminal entities (Roberts 2011). In this sense, as argued by Parry (2006, 2351), embryos move through different representations as potential lives, kin or biological material in part as determined in local contexts.

Building on this notion of liminality as potential lives, kin, relations and research material, we explore the perceptions that couples undergoing IVF have about the embryo made in the process of IVF, and we examine their thoughts, motivations, discussions and acceptability of disposition decisions as potential embryo donors. We aim to demonstrate how perceptions take place within a complexity of societal factors that involve politics, science, medical technologies and IVF processes and practices as well as patient experiences of IVF and religious or nonreligious views and ideas about kin and relatedness. Finally, we explore how the meanings attached to the embryo influence the views and acceptability of its various uses. Importantly, couples in this study were in the middle of treatment so donation of frozen (viable) embryos to research was not yet an option, just fresh non-viable embryos. Equally important is that in Norway donation for reproductive use by others is banned–also shaping the options available. Such regulations and practices existing in Norway will be further explained, as well as identifying the ways in which the embryo was perceived in the Norwegian political context in the 1980s and 1990s. Then, we present how the couples participating in our study conducted in 2019 come to make their decisions and factors influencing their perceptions about the human embryo. According to the results of our study, we suggest that a shift from a perception of the human embryo as life at conception towards a perception of the human embryo coming into being during implantation is taking place—a perception that, in its wider context, most likely holds implications for disposition decisions and intentions.

## Methodology

This article results from the ethnographic study, *Assisted reproductive technologies and epigenetics: an ethnographic study of prospective parents’ experiences, perspectives and imaginations in Norway*, which aims to understand the experiences, perspectives and imaginations of prospective parents about contemporary assisted reproductive technologies (ART), practices and treatment within Norwegian society. This ethnographic study is part of a wider project, *Epigenetics and bioethics of human embryonic development*, and this particular study draws primarily on in-depth interviews and observations of couples currently in treatment for infertility in a hospital setting. Throughout 2019, twenty-five couples were interviewed about their experiences and thoughts on issues relating to childlessness; infertility; reproductive technologies, care and treatment, including how they experience and perceive the status of the embryo and the disposition decisions and intentions. Five health professionals working at the clinic were also formally interviewed, along with conversations throughout the research period.

Eighteen couples were interviewed once; these couples had different socioeconomic statuses and ages (twenty-five to forty-five years), had various cultural backgrounds, ethnicities, and sexual orientations (including three lesbian couples), and were in different stages of treatment. These interviews lasted between one-and-a half and three hours, and both partners accepted the invitation each time, except for one couple, in which the woman decided to participate alone. Seven couples were observed throughout their first IVF cycle, from the initial consultation to embryo transfer; however, all seven couples did not reach embryo transfer because treatment was disrupted for various reasons (spontaneous pregnancy, medical or personal reasons). In-depth interviews with the couples were conducted at the beginning of treatment and after embryo transfer.

Observation of patients during treatment allowed the researcher to follow over time how the couples talked about and related to the process and how they talked about and reacted in practice to making embryos outside of the human body, the fertilization process, transfer, cryopreservation and embryo donation. In addition, the interviews allowed for in-depth conversations about the moral status of the human embryo and about how the couples thought, viewed and talked about the embryo made in vitro. Interviewing the couples together allowed for discussions between the two, as many of the questions posed were not necessarily something the couple had discussed extensively or even agreed upon. Topics related to the human embryo, such as how the couple felt about making embryos outside of the body, whether they perceived these embryos as life, or when the embryos were considered life or human, were discussed in various ways throughout the interview. We asked the couples to describe how they visualised the embryo. Other topics included the technology of cryopreserving embryos and embryo donation to research. We discussed gamete donation more broadly, including embryo donation to other couples, which also provided insights into ideas relating to kinship and family formation. All these topics shed light on the perceptions and views held by the couples about the human embryo, its uses and the topics of research and donation.

The interviews with patients were tape-recorded and transcribed, and field notes of observations were recorded and organised. Health personnel at the IVF clinic received information about the project, and none objected to the presence of researchers during consultations at the clinic. All couples and formally interviewed health personnel gave their written informed consent. Ethical approval was obtained from the Norwegian Centre for Research Data.

The process of analysing the data emphasised descriptions of contexts, underlying meanings, patterns, or themes emerging from the data (Angrosino 2007). Reading and re-reading of the transcriptions and notes on observations enabled the researchers to familiarise themselves well with the data and capture the context of people’s experiences and views (Parry 2006). Central in ethnography, the analysis included and considered different analytical perspectives and comparisons, existing literature and interpretations of others (Angrosino 2007; Stewart 1998). The findings for this study, thus, must be seen in the context of the history and politics of biotechnology and embryo research and reflect how the perceptions of embryos and embryo donation have been conceptualised in other countries around the world.

## Embryo Donation, Research and Regulation in Norway

### Law and Clinical Practice

During the IVF process, several eggs (usually) are harvested from the woman’s body. Not all are mature, and not all will fertilise; for the ones fertilised, some will stop developing or be of poor quality and thus not transferrable (i.e., replaced into the woman’s uterus). In Norway, only one embryo is usually transferred at a time. In selected cases (e.g., high maternal age which may reduce chances of success), a maximum of two embryos may be transferred. Non-transferred embryos of satisfactory quality are frozen for later use.^1^

During treatment, the couples are asked to donate their nonviable fresh embryos that are of poor quality or that cannot be transferred for research. As these cannot be frozen or used for reproductive purposes, the alternative to research is to discard. If the couples have frozen embryos when the limit for cryopreservation ends (in 2020, the limit in Norway increased from five to ten years, or when the woman turns forty-six years of age), they might be asked to donate these surplus frozen embryos for research. Other options are to transfer them back before the legal limit ends or to discard the embryos. The couples in this study were in the middle of treatment, so frozen embryo donation to research was not yet an option for them, but it was discussed in order to grasp their views and acceptance about the different options, choices and uses of their embryos. The couples were invited to donate surplus embryos to research for the purposes of increasing understanding of the development of the human embryo and to improve clinical practice. During this study, the couples were asked to donate their nonviable embryos specifically to a research project on epigenetic reprogramming of early embryo development that aims to develop better tools for embryo selection. The couples received a written information and consent form to be signed before donating embryos. Creating embryos only for the sake of research is prohibited in Norway, so communities using embryos in their research rely on the provision of donated embryos. Thus, the embryos have two different potential representations: as a human being or as a means of improving future reproduction (see Melhuus 2015 for a distinction between reproductive/regenerative capacities); these possibilities represent the liminality, discussed previously, of concurrently being a potential human being and a possible research entity.

In Norway, donation of embryos for reproductive use by other couples has been prohibited, and it remained prohibited during changes to the Norwegian Biotechnology Act in 2020, in which the parliament passed a bill introducing several major changes to accessibility to infertility treatment and legalised new methods (e.g., egg donation and IVF for single women). Despite the ban on embryo donation to other infertile couples in Norway, reflections and thoughts on this were discussed in our study. Reflections and intentions may not always correspond with actual decisions in real life, though, as reported elsewhere (de Lacey 2005; Newton et al. 2007). Nevertheless, these discussions provide important insights into how couples talk about their embryos and how their views are connected to IVF practices and the wider social context in which these discussions take place. The Norwegian Biotechnology Act allowed embryo research in 2007, long after the first political debates began when IVF was introduced in the late 1970s. To shed light on the possible changes in the perception of the human embryo, we reflect on the first debates about embryo research in Norway that occurred in the 1980s and 1990s.

## Life Debates and Embryo Research in Norway

### What Is an Embryo?

Since IVF became available and the technological possibilities expanded, research on fertilised eggs became the subject of political debates in the 1980s and 1990s. At the heart of the debate was the moral status of the human embryo; what is it exactly? Is it a defenceless human being or a lump of cells? Is it something in between (Sirnes 1996, 213)? These views differ, and it is exactly where to draw the line between a fertilised egg, an embryo or a fetus that becomes important in the debate about the human embryo (or fertilised egg) as a human life or not (Melhuus 2015). How we conceptualise or relate to the fertilised egg or embryo might have implications for what we allow or accept as the different uses of embryos made in petri dishes and for how the prospective parents think about or act towards them.

To demonstrate how the status of the embryo is relevant to how research laws are established, Sirnes (1996) compared the debates about research on fertilised eggs or embryos in Parliament in the United Kingdom (U.K.)—the country where IVF was first developed—with those in the Norwegian Parliament (the Storting) during the 1980s and 1990s. The debates were fundamentally different, and the outcomes differed as well; the debates revolved mainly around whether the fertilised eggs constitute life or not, a determination that may or may not justify using them for research. In the United Kingdom, the term *pre-embryo* was introduced and appeared to distance the fertilised eggs from a fetus. The pre-embryo was neither human nor the beginning of a human; rather, it was prior to a human status. Those who wanted to allow research on fertilised eggs argued that before fourteen days (and the formation of the primitive streak) the egg could split into two individual embryos or join together again to form a single individual, and that at this time the pre-embryo forms both the placenta and the future embryo. Therefore, the primitive streak was the individualization itself (Sirnes 1996, 231). Sirnes (1996, 257) also argues that the term pre-embryo acted like a mental door-opener in political debates in the United Kingdom, unlike in Norway. It worked to alter the fertilised egg from consideration as a divine institution of life to consideration as a molecular (cellular) structure and, as such, a potential research entity.

In Norway, the debate turned out differently, and the fertilised egg was described as incipient life. According to Sirnes (1996), the role of science was much more modest than in the United Kingdom and did not represent an alternative ontology to religion. The fertilised egg was not separated from its succeeding stages, so there was no creation of a theology-free space for the first two weeks of the fertilised egg’s existence (Sirnes 1996, 240). Sirnes (1996, 234) also argued that, in Norway, individuality was not a central criterion. Instead of emphasizing the unique individual, as the U.K. debate did, the Norwegian focus was on a common human value: that all fertilised eggs had the same capabilities for human life, which had to be protected. Such views of the embryo explained why Norway maintained a ban on embryo research until 2007.

In 2007, however, the status of the embryo did not necessarily change substantially, but more pragmatic arguments were presented (Melhuus 2015). During the debates and hearings that led to the changes in the Biotechnology Act, respect for human life and the consideration of human dignity were emphasised in general; some conservative and religious institutions referred to the term *incipient life*. The main arguments in favour of embryo donation focused on how Norway already used methods developed by research on surplus fertilised eggs and consequently should contribute to such research; on how research on fertilised eggs in the future could lead to major advances in the treatment of serious diseases for which we currently do not have good treatment options; and how fertilised eggs that could not be used would otherwise be destroyed^2^. Flatseth (2009) argues that the concept of *surplus* fertilised eggs presented in these debates seems to represent what Sirnes (1996) had argued was lacking in discussions in the 1980s and 1990s. The production of surplus fertilised eggs offers a scientific construction that could compete with the concept of incipient life as sacred (Flatseth 2009).

In 2020, the Biotechnology Act was revised for the first time since 2007. The revision allows, by clarifying the interpretation of the law, gene editing research on human embryos under the conditions that they are not transferred to a woman’s uterus and that such eggs are destroyed within the fourteen-day research limit. It was argued that the restriction put unnecessary limitations on research^3^. The majority of representatives from the Norwegian Biotechnology Advisory Board argued that these eggs were already donated to research and could provide knowledge about fertilised egg development that would be valuable not only to IVF technology and success rates but also beyond reproduction to fields such as cancer research (Halvorsen and Borge 2018).

Since the first discussions of research on embryos in the 1980s and 1990s, the political debates about the fertilised egg or the human embryo have changed along with technological developments, and a changing global landscape of biotechnology also has influenced Norwegian political debates. Laws have shifted from banning embryo research on the premise of incipient life to allowing gene-editing research on human embryos predominantly on the basis of scientific arguments. The fertilised eggs thus have emerged to become exactly a liminal entity, one that is between a potential human being and a potential research entity.

## Embryo as Not yet Life

The past decades of IVF technology have paved the way for lab-made embryos and embryo research, and the status of the human embryo has been intensely discussed among politicians, theologians, bioethicists and scientists. Yet few have explored how couples undergoing IVF conceptualise their embryos in a Norwegian context. In the late 1990s Roalkvam (1998) described that the fertilised egg in Norway was seen as incipient life and that discussions about the freezing technology asked what exactly was put in the freezer. Roalkvam (1998) also noted that couples undergoing infertility treatment found it problematic not to be able to give life to the embryos in the freezer and reported that one woman expressed that not giving the embryo life would be as emotionally difficult as having an abortion.

In our conversations with couples undergoing IVF some 20 years later, the views about the fertilised eggs or embryos seemed to have changed. In discussing when life starts and when the embryo is viewed as human, the majority of the participants in this study did not yet dedicate human life to the embryos made in petri dishes. The views ranged from embryos being something alive or something with potential for life to living cells or references like *amoeba*, *bean*, *lump of cells* and *piece of DNA*.

Silje was in her first IVF cycle with her husband and was one of the participants who reflected that she saw the fertilised eggs as something alive: “It is a living cell. It is something alive that is placed inside me and hopefully develop into a human being.” She also pointed out that the IVF makes the fertilisation process more visible and that she knows that the embryo is conceived and somewhat alive when it is transferred to her uterus. Silje and her husband contrasted this experience with their first pregnancy, in which they did not find out about the pregnancy before week ten. Although the embryo was something alive, Silje did not yet consider it a human being, as it only had potential to start developing into a human being after being implanted. Like Silje, Ella, who was in her second IVF cycle, also ascribed some form of life to the embryo she had recently transferred. When asked if she thought of the embryo as human or life, she responded, “I do [consider it life]. The little embryo I carry now, I have talked to it all day long.”

Her husband Eirik looked surprised and did not agree. He answered, “Oh no, I am not quite there.” Ella followed up, laughing and saying, “I really want it to attach to the womb. I have said we will be good parents [laughter]. I know it is irrational.” She ascribed some form of life to the embryo that had been transferred, yet she also placed a lot of emphasis on the implantation process and prayed that the embryo would attach.

Johnny and Mathilda, who were in their first IVF cycle, also embarked on a discussion of what constitutes human life and when an embryo becomes a fetus or a baby. Johnny explained his view on the topic:

There is potential for life at least. By definition, it is life since it is cells that are alive. But we kill cells when we eat food for example, so why are these cells suddenly much more valuable than other cells? I think it is not human until they grow in someone's belly and become a human being who is born.

Mathilda followed up by also referring to the IVF process:

It is very difficult to define when it goes from being an embryo to becoming a fetus to becoming a baby. It is a gradual transition...but when it comes to the embryos in the lab, I do not think of it as a baby. It is the precursor to what can become a baby. For my own part, there is some psychological protection in the process.... I cannot begin to think of an embryo that did not survive thawing as a baby who died, then I go crazy. I have to think that embryos are embryos, a blank canvas or an empty starting point. It is only when it grows inside the womb that it can develop into a baby.

Mathilda and Johnny adopted the gradualist view of the development of the embryo and fetus and noted that not looking at the embryos as potential babies is a way of protecting against the emotionally difficult state of IVF treatment. Like Ella and Silje, though, they attributed growing inside the womb as a point of reference for determining some form of human life.

Anita, also in her second IVF cycle, was clear in her views about the embryo. When describing if she saw the embryo as human life, she said:

No, the embryo cannot make it on its own. It can only develop to be something after implantation in the uterus. There, life really begins to develop. Before that, when it is just an embryo and not yet implanted, it is just a lump of cells. It is a mix of two people, but it is not viable until it has come a step further.

She also referred to the implantation process and explicitly saw the embryo as a lump of cells before implantation. Sofia, who was in her first IVF cycle with her husband Pierre, was also clear that implantation is her point of reference when discussing life:

Not to me before it is on the inside of another life…or when it has implanted, when you know it has attached to the womb in a way.... Those two weeks are an uncertain phase…. You cannot walk around and think you are pregnant the first two weeks, then you go crazy…. It is nothing until it is proved it is there.

Pierre was a bit more reflective on the topic and did consider it something living:

I feel that it's more like living processes, not necessarily life, if there is a difference. But some living processes that happen to a greater degree than it would if it just happened inside you [his wife] for some strange reason. Because it gets so visible, it's kind of taken out of some weird context. But I do not think there is life immediately.

Pierre points to an interesting aspect of IVF, the fact that embryos are made outside the body and as such are made visible, yet at the same time they are not given the status of life. Such views were also made clear in an interview with Lene and Peder. At first, Lene was quick to provide her view of the embryo as life or not: “To be honest, I do not think so.” Peder followed up by stating that it is a “piece of DNA in a dish.” Lene continued by explaining, “For my part, perhaps when you know that you are pregnant and are over the uncertain phase…at the legal limit of induced abortion. In that period, I do not consider it life or a baby.” She also explained, though, that “when I think about it a bit more, maybe it has some potential for life, but when you hear the heartbeat, or when you know the heart beats, you can hear that quite early. If I had heard that, I would think that it was life.”

The couples, in various ways, demonstrated how implantation and a confirmation of the attachment of the embryo to the uterus wall were for most deemed the start of life—but not necessarily deemed a human being. As Lene noted, knowing or visualizing the heartbeat through ultrasound was for many the strongest confirmation of human life, although exactly when the heart starts beating and when you can visualise it were often unclear. Others referred to the importance of being past the uncertain phase at week twelve and pointed to that time as when the miscarriage risk decreased and as the legal limit for induced abortion in Norway. As such, conceptualizations of life and when it becomes human reflect a gradual process linked to medical technologies, such as pregnancy testing and ultrasound, and to political factors. Furthermore, descriptions by the couples of the fertilised egg or embryo as a cell that has divided a few times, as something living that has potential but is not viable and is not yet life, speak to scientific understanding rather than religious thought. With a few exceptions of Catholic and Muslim beliefs and one woman who expressed some agnostic thoughts, none of the participants defined themselves as religious. Most participants were concerned with demonstrating that they did *not* have a religious affiliation. Arguably, their description of the embryo as a cell provides a competing discourse, a scientific construction that can compete with the construction of incipient life as sacred (cf. Flatseth 2009).

Given Mathilda’s and Sofia’s quotes about the context of infertility and IVF, to grant the embryos human life or to think of them as babies is too difficult in a process that often involves several failed attempts of becoming pregnant. The IVF process makes visible the fertilization process, and not granting life to the embryos immediately is a way for parents to emotionally protect themselves. Couples consistently pointed out that the waiting time between the transfer and the pregnancy test was particularly tough. A few attached some form of (potential) life to the transferred embryo, but the confirmation of pregnancy was for most the start of life; the uncertainty of the result of treatment at that time was reduced (despite entering an uncertain phase in terms of miscarriage risk). Furthermore, the language used in IVF not only includes a medical vocabulary such as cell division and blastocysts, but also focuses on implantation and attachment to the womb. That phase cannot be controlled, as reflected by language such as “fingers crossed that it will attach this time” or, when successful, “on the second frozen embryo transfer, it attached.” Arguably the view about the embryo is influenced by the IVF process itself and, for many, recurrent failed attempts and experiences that life is not life until implantation is confirmed, and milestones that are shaped by science and politics.

These views about the embryo as not yet life, are connected to discussions about embryo donation for research, which is discussed in the next section.

## Embryo Disposition Options

### Research, Discard or Transfer Back?

All the couples in our study chose to donate surplus embryos of poor quality for research; these embryos were considered unqualified for implantation into a woman’s womb. Importantly, these embryos were already designated as nonviable, donation seemed acceptable because of the poor quality and the inability of couples to use the embryos for IVF. In discussions about the frozen embryos, the vast majority decided that donating to research was better than discarding. If the participants had reached the number of children they wanted, uterine transfer just for the sake of not discarding or not donating to research was not an option for most, nor did they express a wish for an alternative ceremony. Except for one woman who considered transferring the best option but pointed out that she would have to discuss this idea with her religious leader to make sure that her choice aligned with her religion.

While the nonviable embryos were accepted donor items, disposal of the frozen embryos required a few more minutes of reflection for some individuals or couples. What sets these embryos apart is that frozen ones have a potential. The immediate thought, however, of having frozen embryos was not about having “life” in the freezer. It was not described as ethically challenging, but rather seen an opportunity for pregnancy and not having to go through hormone stimulation and egg retrieval again immediately. For some, to think of a potential child in a freezer was bizarre, like science fiction, yet often linked to humour and laughter. One participant noted that “they must be really cold, how sad being in a freezer, they’d be much better off in a warm uterus.” Another participant noted that she saw the frozen embryos as not yet life, yet a potential life “on hold” due to its frozen state. A few worried about the consequences for the child’s health, but the majority of participants expressed fascination with the technology and scientific developments and saw the developments as representing hope and potential.

In our discussions about donating embryos to research, we first discussed the nonviable embryos and how participants felt about being asked to donate these to research. Anita, who was in her second IVF cycle, said:

It is given to research and they can find out more about this process and how things are; therefore, I think it is important to contribute. Rather than just becoming medical waste, it’s better that someone can use it to learn something.

Her husband Ola followed up and confirmed her view:

Use it, do *not* just throw it away.... It is the least you can do as long as you use all this [IVF treatment]. I want to contribute to moving forward, to find out more, to learn. If everyone says no and throws in the trash, it’s silly, then you throw away so many opportunities to learn and to help people…. If some of what we do not need [during the process] can help someone else one day in the future, and maybe they will get the child they wish for, that is positive.

When asked if there was any difference in their thoughts regarding the frozen embryos, Ola replied:

I don’t think there is any difference…the moment we do not need them anymore…maybe it is even better that they get to research them, because then there is perhaps better material to research in order to get better results out of it, so there is no reason not to let them research it.

To agree to donation when the embryo was considered unneeded was common, as was the idea of using them and learning from them. Such ideas were also something Clara and Nils talked about. They already had one child through IVF and were now undergoing treatment for a second child. Clara was quick to reply that they had decided to donate their nonviable embryos to research: “Yes, we donated to research. If we don’t, then we couldn’t be here and receive the help and treatment we get now.” Neither remembered what kind of research they donated to, and Clara followed up by stating, “In Norway, things are so strict, I am a little naïve. I am not that critical of what you donate to in Norway.” Clara had the same thoughts about donating frozen embryos; she did not consider these embryos as life yet and considered them even better research entities as these were successful in contrast to the nonviable ones.

Not remembering exactly the donation placement was common among participants, and they did not wish for more details about the donation. Sofia shared that she trusted Norwegian research and supported research to improve IVF treatment and the scientific understanding about failure to conceive to help couples have the child they want. As long as the embryos were not used by them, she approved of donation to research. For her, there was no difference between nonviable embryos and frozen ones, as she did not view them as life yet. Her husband Pierre needed a moment of reflection. Concerning the nonviable embryos, he agreed, because they could not become a pregnancy. On the question of the frozen ones, he said: “There was something that is somewhat different when I thought that this is our potential child…. It is a little fuzzy, but I agree, I had probably decided that it's okay, yes.” Sofia confirmed and continued, “Yes, because it will not be used by someone else, it will be used for something positive.”

To attach their view of research to the embryos as not yet life was common. Also, pragmatic arguments—such as wanting embryos to be useful and not to be wasted—were frequently heard when discussing donation to research, if the embryos would not be used by the couples themselves. The nonviable embryos thus were unproblematic to donate. The frozen ones represented a stronger potential for life for some, but first and foremost referred to as a possibility to get pregnant and as a way to postpone the stress and pain of hormone treatment and egg retrieval. However, if the embryos were no longer needed, donating to research was predominately seen as the most attractive option, and discarding them was perceived as wasteful. This decision was made in the context of trusting the Norwegian research environment not to do anything suspect with the embryos. Furthermore, the couples hoped to give something back in gratitude for what they received, in solidarity with other couples in similar situations, to help involuntarily childless people in the future. The donation of embryos to research is their way of contributing to scientific developments in the field of reproduction and IVF. However, when discussing embryo donation to other couples, the meanings attached to the embryo changed.

### What About Donation to Other Infertile Couples?

Embryo donation for reproductive use by others is not legal in Norway, but couples were open to the possibility of legalizing it for those who would prefer this option. For themselves, however, the vast majority of participants found it difficult to consider donating their embryos to other couples. The pre-implantation embryo was seen as cells, or living cells or potential life, and if not needed, as possible research objects. But potentially available for donation to other infertile couples, the language about the embryos frequently changed from *cells* to *our genes*, from *embryo* or *potential child* to *our child* and *sibling*.

Sofia’s comment when discussing embryo donation to research was, “Yes, because it will not be used by someone else, it will be used for something positive.” This statement indicates that she had rather negative connotations about embryo use by someone else. Sofia stressed that donating to another couple is different: “It would have been strange to adopt away a child you haven’t yet gotten.” When discussing embryo donation to other couples, such views were widespread among the participants in this study. May, who was in her first IVF cycle with her partner Liv, pointed out that embryo donation should be legal but does not believe she could do it herself. Liv followed up by emphasizing that children from those embryos “will be 100% siblings with our children”. Such a focus on siblings was also expressed by Hedvig, who was in her third IVF cycle: “That is a terrible thought, because then you make a firm choice on behalf of the already-started individual and their siblings. Imagine if you had been told that you have a biological brother who had grown up somewhere else!”

Hedvig’s husband responded, laughing, “I was thinking, oh how nice, thanks for the help,” but concluded, “Oh my god, no,” after listening to his wife. Evelyn and Simon also discussed the problem of siblings. They already had a child before entering IVF treatment, and Simon argued that he personally would not want to donate but that it should be legal for those who want to. Evelyn followed up on his comment by saying:

It is a bit counterintuitive for me personally. There are a lot of good arguments for it to be allowed. But it’s weird, then X [their son] has a sibling, 100% his sibling, who grow up somewhere else, with another family which he has no contact. Is it actually our child? Biological with our genes and our appearance, and who looks like him. And it is very strange that it should be a completely foreign family.

While considering the problem of a “100% sibling with our child,” they also pointed to biology and genes, a topic discussed by other couples. Clara noted that she thinks donation should be legal and that the couples should be given the choice to donate or not, but she found it difficult personally: “I think it is totally fine if someone wants to [donate], but that is not to say that I could. It is in fact a fertilised egg that *is us.* I don’t think I could.”

Clara also noted that she feels cruel when refusing to help others by donating their embryos since they receive help through IVF, but the thought of donating their embryo was just too difficult. Such perceptions of the embryo as being them were also discussed by Maja and Petter. They agreed to donate to science but not to another couple. “We agreed to donate surplus eggs to research, which is fine,” Petter said. “I am not that keen on donating the embryo to another couple. It is mine, or our genes,” Maja noted, before Petter continued, “Yes, a complete set of genes. That is very difficult.”

Such references to a “complete child” were also noted by Silje, who hesitated but was not completely negative; she strongly believed that everyone should be allowed to experience becoming parents. Although her husband found the thought of it just too awkward, she reasoned:

I do not know. If I had felt absolutely done. I must have known who it was, I think…because there are so many weird people. But I wouldn’t know too much either, but only that they were good people…. It had been sort of completely our child [helt vårt barn], that had grown up somewhere else.

The few who did not completely cut off the thought of donating their embryos discussed it in the context of solidarity, of helping others who struggle to conceive. It was also discussed in relation to the best circumstances; of donating to family so the child would stay within the family or at least so the parents would know who the receiving family is, or anonymous donation so that the activation of kinship relations would be somewhat cut. No matter what they would finally do if confronted with the choices, the participants in these discussions demonstrated the meanings attached to embryos and ideas of kin and relatedness—and the idea of what *our child* actually means in such a context.

Descriptions of *our genes*, *a complete set* and *our child* were common, and the concept of the child as a *100% sibling* was commonly presented. The embryo is seen as *theirs* genetically, a combination of the two and a genetic uniqueness that represents them as a couple, as well as a potential sibling to the couple’s existing or future children. As demonstrated by Liv and May, who as a lesbian couple had donor sperm, they focused specifically on the child as a *100% sibling*, and also emphasised the embryo as *our child*, despite the fact that one of the women where not genetically linked to the child. While not focusing on the *complete set* of genes, the child were still *theirs*, also demonstrating how people adjust the meaning placed on genes and gestation when establishing kin and relations in alterative family making (Thompson 2005). If the status of life is granted to the embryo at implantation, topics such as genetics and potential siblings gain importance when embryos are activated as a potential human being in a(nother) woman’s uterus, and thus a potential kinship relation begins.

## Discussion and Conclusion

Expressed in various ways, couples in this study embedded in a Norwegian context do not perceive their pre-implantation IVF embryos to be human life yet and rather dedicate the start of life to a successful implantation. This view of the pre-implantation IVF embryos as not yet life indicates a shift in the perception of the human embryo or the fertilised egg from incipient life—human life at conception, a dominant concept in the discussions of embryo research in the 1980s and 1990s—to potential life or life represented by implantation in the woman’s uterus and a positive pregnancy test. In the 1980s and 1990s, the role of science was modest or absent in the political debates about the status of the fertilised egg (Sirnes 1996), whereas, in our study, science seemed to contribute to the dominant understanding and view of the fertilised egg or the human embryo as not yet life. The role of science is visible both in the use of scientific arguments in political debates leading to the legalization of embryo research in 2007 and onwards and in the way that couples currently using IVF technology described their views of the human embryo. The couples consistently visualised the embryo as a cell, typically describing it as a circle with circles inside, a picture of the fertilised egg commonly used in IVF. Science is visible through the language of living cells and pieces of DNA and in the way the couples relate to science and research in terms of trust and hope in what IVF technology can provide. The changes in viewpoints seem to have taken place along with increased knowledge about biological processes and IVF technology itself, and, bearing in mind that most participants in this study did not have religious convictions, along with the consideration of science as a competitor to religious connotations.

It seems there is an increased scientific and molecular (cellular) representation of the human embryo, which is also discussed in Flatseth’s (2009) work, who further argues how the notion of fertilised egg cells works to emphasise that we are talking about cells; its plural representation also draws attention away from the individual (Flatseth 2009). Thus, a scientific view of eggs and sperm, fertilised eggs and embryos influences the way we see the embryo that is made outside of the body. According to Melhuus (2015, 131), the embryo is removed from its localized origin and pictured as something out there and not internal to the body. Outside of the body, the embryo exists in a liminality, as a potential research entity and a potential kinship entity, as pointed out by Franklin (1995), exactly in a contested location between science and nature. The embryo is not yet a human life, and it is somewhat a microscopic entity with the potential for both research and pregnancy. Being on hold, as a participant said when describing the frozen embryo, illuminates the liminal existence, the either/or state of the embryo.

When the embryo is transferred, it is something living, with the potential to become life or not. When implanted, and when pregnancy is confirmed, the fertilised egg or embryo is granted some form of life and thereafter gradually becomes a full human being. As such, the process is seen as continuous and gradual, as also described by Flatseth (2009). How medical technologies such as ultrasound influence our perception of the fetus as an individual is commonly argued (Ravn 2004). In our study as well, the definition of life and the move towards a gradual status as a human are linked to technologies, biomedicine and politics. Importantly, however, as pointed out by Krones et al. (2006, 18), there is probably a cluster of attitudes in society, of opinions on the beginning of human life, on general values and on religious feelings that might be facets of different ideas of the human. In their study, the majority of the German population, as well as couples who experienced IVF and created embryos in vitro, saw implantation of the fertilised egg into the uterus as the crucial process that transfers the biological entity into human life (Krones et al. 2006). According to Roberts (2011), most North American and Western European couples undergoing IVF believe that the transfer of an embryo to a woman’s uterus activates the possibility of an individual; such belief is also present in Israel (Raz et al. 2016). Our study included similar viewpoints. Investigation of the viewpoints of the Norwegian public—not only those influenced by being in the middle of the IVF process themselves—would be interesting. Importantly, IVF couples have often, through many failed attempts, experienced the lack of life in an embryo when it does not implant and lead to pregnancy. These experiences arguably influence the way the couples speak about and relate to the pre-implantation embryo.

Such views of the pre-implantation embryo and the tendency to dedicate the start of life to a confirmed pregnancy shed light on why donating embryos to research is rather unproblematic or at least accepted, whereas the thought of donating to other infertile couples is perceived as problematic and difficult—as kin relations with the embryo starting in another life. When the embryo is implanted in a woman’s uterus and pregnancy is confirmed, human life activates and the embryo becomes a potential kin. Thus, to become kin it must implant; then mothers, fathers, children and siblings are created. These definitions of kin define social relations and are central to a couple’s understanding of what constitutes family, relations, and responsibilities.

As our study demonstrates, when couples discuss donation to others, the embryo is considered *a full version of us*, *100% biological siblings to our children*, *our genes* and *our child*. The unique combination of the genetic material of the two parents representing the embryo is significant, also reflecting the genetic criteria of kin relatedness in a Western context (Franklin 1995). However, when the embryo is donated to research, this genetic composition is not of importance. Put differently, although the couples think that embryo donation to other couples should be legal, the problem of donating to others reflects the potential for full biological siblings to their future or existing children and represents a complete set of genes from the couple, which could develop into a life by implementation in (another) uterus. The potential relationships based on genetic relatedness and the implications of having a full genetic child growing up with another family characterise the reflections surrounding the donation choice. The lesbian couples using donor sperm argued the same way about donating to other couples. For them, it was primarily about siblings, but also the idea of the child as *theirs*, despite missing the genetic link to one parent. It does not mean that the genetics are not of importance, but the meaning placed on genes is adjusted when establishing the child as family and kin.

Constructing the embryo as kin and thus making it problematic to donate to others has also been documented elsewhere (Goedeke et al. 2017); in New Zealand, the importance of such genetic connections carries ongoing social ties that have implications for embryo donation to other couples (Goedeke et al. 2015). Issues of full genetic relatives or a full genetic sibling has also been discussed in studies such as de Lacey (2005), McMahon et al. (2000) and Paul et al. (2010). Millbank et al. (2016), however, show that embryo donation was a desired option among their respondents in Australia; in contrast to what has been previously suggested about donation as difficult and the least preferred or as avoidance of the worst outcome (Hammarberg and Tinney 2006; de Lacey 2007). In explaining this contrast, Millbank and colleagues (2016) point to different regimens of embryo donation and explored how laws and policies shape experiences but also noted that donors in their study did not prioritise genetic relatedness in their experiences of kinship. Both New Zealand and Australia allow embryo donation to infertile couples, yet there are different legal and counselling regimens. Interestingly, in a study in Sweden conducted when embryo donation was not allowed, nearly three-quarters of infertile couples with surplus embryos were in favour of embryo donation to infertile couples (Wånggren et al. 2013). While their study acknowledged that such views might change when confronted with the choice in real life, our study also showed that the majority were in favour of allowing embryo donation *in principle*, but that did not mean that they prefer it themselves. Laws and policies, and what is possible or allowed, might affect people’s preferences, thoughts and actions. The couples in this study, however, had not given embryo donation to other couples a lot of thought, as they were in the middle of treatment using their own embryos. Yet, they had strong views on how they would act towards it; supporting its legalization, but not willing to donate themselves.

In our study, the embryo’s status as life at implantation, together with a biogenetic understanding of kinship, sheds light on why embryos are preferred given to research rather than to another couple. The state as not yet life before implantation and the consideration of implantation as activating human life contribute to the liminal existence before transfer and reinforce the embryo as a possible research entity. Donating to research as the best option is also understood in the context of science, trust, solidarity, reciprocity and pragmatism. To donate spare embryos to research, deriving from a reciprocal obligation to give something back, was also noted by participants in a study by Fuscaldo and colleagues (2007). Donating in the context of a desire to help others and advance scientific and medical knowledge has also been shown by McMahon et al. (2003), although such solidarity and reciprocity have been problematized as if feelings of indebtedness towards the clinic helping them may make people donate to research to give something back (Scully et al. 2012). In our study, IVF users strongly supported increased scientific knowledge about human reproduction and improvements to IVF technology, making discarding surplus embryos wasteful. It has also been argued that a perception of waste must be seen in the context of the efforts involved in making them (McMahon et al. 2003; de Lacey 2007; Provoost et al. 2009). Such pragmatic arguments of not wasting material were also prominent in the discussions about allowing embryo research in 2007; some aspects of citizens’ views about IVF practices seem unchanged today.

How IVF couples relate to their surplus embryos in different global/regional contexts has been extensively discussed. In different local contexts, embryos have different representations as potential lives, kin or biological material, as noted by Parry (2006). As the literature suggests, the choices people make on this topic are often based on grounds beyond religion or science. As our study indicates, these choices are strongly relational and are also shaped by ideas of kinship and relationships. Roberts (2011) has shown that, in Ecuador, the embryo is considered an integral part of family, and frozen embryo donation constitutes abandonment. Consequently, these patients prefer to discard embryos rather than freeze them for a future away from their relations. It is not life that is central but the group that would abandon one of their own (Roberts 2011, 232). In our study, we argue that, along with a persistent understanding of the significance of kinship, the embryo becomes a liminal kinship entity as described by Franklin (1995); it is not yet human life but holds the potential for important relations between the couple (as a representation of them) and between siblings. Before implantation, though, the embryo is a tiny, cellular entity in a lab that can be donated for research; it is not yet life but has the potential for life. Interestingly, such views might support expansion of biotechnology research on the pre-implantation embryo, and the couples in this study also persistently supported the legalization of embryo donation to other couples. Yet as shown, when implanted it becomes kin; and mothers, fathers, children and siblings are created, making such donation regimes difficult in practice. As such, the reflections on the human embryo and disposition choices our study have identified work on the interface of science and technology, of politics and religion, of IVF experiences, and of kinship and relations.

## Footnotes

1 The terms fertilised eggs and embryos are used interchangeably, although there is a slight tendency to use term fertilised eggs when the couples are told how many eggs were fertilised and to use the term embryos when they are transferred or cryopreserved.

2 Recommendation and Proposition to the Storting (Innst. O. nr. 62 (2006-2007). Ot.prp. nr. 26 (2006–2007)).

3 Recommendation to the Storting (Innst. 296 L (2019–2020)).
